# Development, validation and reliability of a questionnaire to evaluate coaches’ and players’ perceptions, learning, and resources regarding the ruck in rugby union

**DOI:** 10.17159/2078-516X/2024/v36i1a17109

**Published:** 2024-05-15

**Authors:** M Brooks, N Parmar, W Kraak

**Affiliations:** 1 Division of Sport Science, Department of Exercise, Sport and Lifestyle Medicine, Stellenbosch, South Africa; 2 London Sport Institute, Faculty of Science and Technology, Middlesex University, London, UK; 3 Department of Sport, Recreation, and Exercise Science, University of the Western Cape, Cape Town, South Africa

**Keywords:** Questionnaire, attitudes, behaviours, coaching, injury prevention, learning

## Abstract

**Background:**

The lack of a reliable research tool for assessing the attitudes, behaviours, and learning resources of rugby coaches and players regarding the ruck event is a significant gap in rugby research.

**Objectives:**

This study aimed to adapt an existing questionnaire focused on the tackle event and to validate and establish the reliability of the instrument. The questionnaire explores the attitudes, behaviours, and learning resources of rugby coaches and players, with a focus on the ruck event and its impact on coach and player development, as well as coaching practices.

**Methods:**

A seven-step design process was followed to validate the questionnaire’s content, construct, clarity, and relevance. A panel of 12 experts evaluated the questionnaire, followed by a test-retest procedure involving 15 coaches and 16 players, highlighting the effectiveness of this questionnaire, and emphasising its potential to generate data that can impact the field of rugby coaching and player development.

**Results:**

The questionnaire was deemed appropriate and clear by the expert panel, with an average completion time of 22 minutes. Moderate to good agreement was observed among players (ICC Agreement = 0.71) and coaches (ICC Agreement = 0.88), with high response consistency (ICC Consistency = 0.71 for players and 0.87 for coaches). Significant agreement was also found in Kendall’s W scores (players = 0.85, coaches = 0.93, p<0.01).

**Conclusion:**

This study presents a developed questionnaire noted for its clarity, reliability, and consistency. It serves as a valuable tool for future rugby research, with the potential to impact coach and player development significantly.

Rugby union, commonly referred to as rugby, is characterised by high-intensity physical contact and running, leading to safety concerns primarily due to frequent collisions, especially in tackles and rucks. ^[1]^ The ruck is seen to have the second-highest injury occurrence following the tackle. ^[1,2]^ Despite the importance of these facets of play, there is a lack of research on safety during the ruck. ^[2]^ In the absence of a valid and reliable research tool to assess the attitudes, behaviours, and learning resources of rugby coaches and players concerning the ruck event, a notable gap exists in the current rugby research landscape focusing on coach development, coaching practices, and player development. It is important to address this gap by enhancing coaching resources, as this is essential for effectively preparing players to safely steer the demanding aspects of the game.

The interconnected nature of players’ knowledge, attitudes, and behaviours suggests that education can significantly influence these areas. ^[3]^ Other research has also highlighted the importance of coaches having an extensive understanding of various aspects of rugby, including injury prevention and player welfare. ^[4]^ Therefore, coach education is essential for rugby injury prevention and risk management. ^[3]^ Although safety accreditation courses such as Australian RugbySmart and South African BokSmart have effectively reduced injury risks, ^[5]^ transferring this knowledge to players remains a substantial challenge. ^[5]^ Current coaching resources are often insufficient in preparing players for matches, particularly in critical skills such as tackling, ball-carrying, and rucking. ^[2]^ The discrepancy in these skills between senior and amateur players highlights the need for improved training and skill development. ^[2]^ In junior rugby, coaches’ knowledge, attitudes, and behaviour are pivotal for injury prevention, ^[6]^ underlining the important impact these factors have on young players. ^[6]^ Coach education can include formal training and informal learning through experience and social networks, ^[5]^ yet there is limited research on coaching behaviours in rugby. ^[7]^ Utilising role models or peer leaders in coach education programmes has shown to be effective, ^[8]^ but effectively transferring knowledge from coaches to players is an ongoing issue. ^[9]^ In this context, “attitude” refers to a person’s beliefs about the consequences of specific behaviours, which include on-field actions, interactions with coaches, opponents, officials, and teammates, as well as the use of equipment. ^[10]^ Off-field behaviours, such as diet and sleep, are also important in sports injury prevention. ^[10]^ However, player behaviour, a key element in injury prevention, has not been studied extensively. ^[6]^ Coaches influence player behaviour significantly, emphasising the crucial role of knowledge transfer in sports coaching for athlete development and success. Coaches impart technical skills, tactical awareness, mental resilience, and strategic understanding. Successful knowledge transfer hinges on effective communication, with coaches simplifying complex concepts. ^[11]^ Practical demonstrations and modelling enhance players’ ability to replicate actions, leading to increased success in knowledge transfer. ^[12]^ Challenges arise from individual differences in learning styles, requiring coaches to tailor teaching methods for equitable knowledge transfer. ^[13]^ Environmental factors, such as crowd noise and competitive pressure, can hinder knowledge absorption, prompting coaches to consider these influences in planning and delivering strategies. ^[14]^

Questionnaires are important tools in sports science research. ^[15]^ These instruments, comprising both closed and open-ended questions, are essential for collecting data and providing insights into various aspects within the domain they are being used for. ^[15]^ Their development must follow a meticulous process to meet established quality standards, ensuring validity and reliability before implementation. ^[15]^ Validity in questionnaire-based studies is important for assessing the accuracy, generalisability, and relevance of measurements. ^[16]^ Validity can be content validity, face validity, and construct validity. ^[15]^ Reliability, defined as consistency across multiple measurements, is also critical, ^[17]^ highlighting the importance of producing replicable results using a consistent methodology, like Cronbach’s Alpha. ^[18]^ Furthermore, the reliability of a questionnaire aids in the credibility and replicability of the research and allows for comparative and longitudinal studies over different groups, cultures, and over time.^[18]^ In the context of this study, the primary objective is to adapt and develop a questionnaire that was used in previous research on the tackle event, ^[6,10]^ that not only ensures validity and reliability but also effectively assesses the attitudes and behaviours, and learning resources of rugby coaches and players, focussing on the ruck event.

## Methods

### Research design

The research design consisted of two stages to ensure the appropriateness, validity, and reliability of an adapted questionnaire for assessing ruck-related attitudes, behaviours, and learning resources in rugby. Initially, an existing questionnaire, ^[6]^ originally designed for the tackle event, was adapted to align with the research objective centred on the ruck event (Stage One). The questionnaire by Hendricks and colleagues consisted of four sections: Section A focused on the coach and player’s demographical information; Section B focused on attitudes and behaviours during training; Section C focused on attitudes and behaviours during match-play; and Section D focused on learning resources towards the ruck event. The adaptation was considered as the original questionnaire highlighted attitudes and behaviours towards the tackle event, which aided the researchers in creating a base for the questionnaire. This adaptation involved an extensive literature review and consultations with an expert panel. Stage Two comprised two sub-stages: initially, a cross-sectional semi-structured questionnaire was issued to rugby experts to refine the content and validity of the questionnaire; subsequently, a structured questionnaire was distributed to rugby coaches and players outside of South Africa to evaluate the reliability of the questionnaire through a test-retest method. The study was approved by the Health Research Ethics Committee 2 (HREC2-S22/07/120) of Stellenbosch University.

### Participants

During Stage One, the authors brainstormed, included, and excluded questions from the existing questionnaire, ^[5,6]^ which originally focused on tackle-related aspects. In Stage Two *(A)*, the expert panel (n=12) consisted of twelve representatives selected based on their experience in both amateur and professional rugby environments in South Africa. This panel included two rugby coaches, two strength and conditioning coaches, two representatives specialising in injury prevention programmes, two rugby scientists experienced in rugby-related studies, two rugby players, and two rugby referees with amateur and professional rugby experience in their respective fields. The panel members had an average age of 36 and an average experience of 15 years in their respective fields. This approach was employed to involve rugby stakeholders to ensure the questionnaire’s validity, which was important in ensuring that the questions were appropriate for assessing the ruck event from many expert perspectives. For Stage Two *(B)*, by use of a power calculation conducted by the statistician during the research proposal stage, the minimum number of required participants for each stakeholder group was fifteen (n=15). With the above being mentioned, fifteen (n=15) level coaches and sixteen (n=16) level players from outside South Africa, with an average age of 37, and 21 years of experience for the coaches and an average age of 28, and 12 years of experience for the players, volunteered to participate in the study. They were recruited to assess the reliability of the questionnaire using a test-retest method, ^[18]^ conducted 30 days after completing the questionnaire.

### Data collection procedure

#### Development of the questionnaire

While developing this questionnaire, the study utilised a seven-step scale design approach tailored for educational research. The AMEE Guide introduces a systematic, seven-step method specifically designed for creating high-quality questionnaire scales suitable for programme evaluation and research purposes. This approach seamlessly integrates various techniques commonly employed by questionnaire designers into a cohesive and effective process. ^[15]^ The questionnaire was developed through the adaptation of an existing questionnaire that focuses on the tackle event, following a systematic methodology with the overarching goal of developing a reliable and effective instrument for data collection focusing on the ruck event. The “tackle” questionnaire mirrored the adapted version, encompassing demographics, training, match play attitudes and perceptions, and resources. Adjustments were made to account for the distinctive features of the ruck area: gathering additional demographic information about coaches and players, adapting questions on key factors with two additions, modifying tackle-related questions to align with the ruck, and introducing extra questions in the resources section to enhance understanding of ruck-related needs. This methodical approach ensures that the questionnaire is well-structured, aligned with the aims, and capable of generating valuable and trustworthy data for analysis. ^[19–21]^

#### Validity

Content validity utilised in this study relies on an expert panel’s judgment to assess if the questionnaire effectively aligns with the ruck event. In this study, alongside content analysis, experts with extensive rugby experience evaluated questionnaire sections for clarity, flow, and question appropriateness. A Likert scale was used for Content, Construct, Clarity, and Relevance validation: the expert panel scored each question from 1 to 10, representing ‘not relevant’ to ‘highly relevant’. The validity part of the study comprised two phases: an initial Likert scale assessment by a panel of 12 experts and a second phase involving telephonic interviews with selected expert panel members. During these interviews, we sought clarity and ensured comprehension based on their feedback. Experts completed the questionnaire and recorded the time taken. ^[20]^

#### Reliability

For this study, the guideline for reporting reliability and agreement studies (GRRAS) was followed, ^[22]^ aligning with the Equator Network guideline for this study type. As mentioned earlier the test-retest reliability (stability test) was used to measure its reliability. The intraclass correlation coefficient (ICC) and Kappa coefficient were utilised to measure test-retest reliability and agreement for categorical variables. ^[23,24]^ A convenience sample from the coaches and players who met the inclusion criteria was recruited via social media to complete the questionnaire. Respondents were requested to re-answer the questionnaire 30 days after the initial completion to minimise memory interference. The choice of the 30-day interval was based on previous research, which aims to avoid recall bias while avoiding response fatigue. ^[14]^ Data from both questionnaire rounds were tabulated in electronic spreadsheets (Excel®, Microsoft, 2010), and ICC and Kappa coefficients were calculated based on the nature of the questions, whether unranked (nominal) or ranked (ordinal). ^[25]^

### Statistical analysis

The Centre for Statistical Consultation assisted with the statistical analysis. Statistical analyses were conducted using Statistica software (version 14.0.1.25) with a significance level of 5% applied (p<0.05). Descriptive data for the Likert Scale responses by the expert panels were reported as frequencies (number of responses) and percentages. The Intraclass Correlation Coefficient (ICC) was employed to assess agreement and consistency in continuous or interval-level data. ICC estimates and their 95% Confidence Intervals (CI) were categorised as follows: values less than 0.5 indicate poor reliability, between 0.5 and 0.75 suggest moderate reliability, between 0.75 and 0.9 indicate good reliability and values greater than 0.90 signify excellent reliability. During the proposal stage of the project, the research team and the statistician agreed that only values above 0.70 will be considered. ^[22,23]^ Kendall’s W was utilised for ordinal data, specifically focusing on ranking order and measuring the concordance or agreement among raters or observers when ranking items or subjects.

## Results

The results of Stage 2(B) (validation by the expert panel) are presented in Table 1. Concerning content, construct, clarity, and relevance, all the experts ranked the criteria between 9 and 10 on the Likert scale, indicating high relevance, clarity, completeness, and meaningfulness of the questions. The results reveal that the questionnaire was clear, they did not experience any doubt regarding the questions and the mean time to complete it was approximately 22 minutes.

Table 2. presents the ICC scores assessing players’ and coaches’ agreement and consistency. The ICC Agreement score for players is 0.71, revealing a moderate agreement among the players’ responses. Additionally, the ICC Consistency score for players is 0.71, indicating a moderate degree of response consistency and reliability. On the other hand, coaches demonstrate a notably high level of agreement, with an ICC Agreement score of 0.88, signifying a good agreement in their responses. Furthermore, coaches exhibit a good degree of response consistency, reflected in an ICC Consistency score of 0.87. Considering the cut-off point of 0.65, all three sections of the instrument were deemed reliable.

Table 3. displays Kendall’s W scores for players and coaches, demonstrating a notable level of agreement in their responses. For players, Kendall’s W score is 0.85, indicating a strong agreement (p<0.01), while coaches exhibited an even higher level of agreement with Kendall’s W score of 0.93, also deemed statistically significant (p<0.01). This highlights a strong agreement within both groups, affirming the reliability and consistency of their perspectives.

## Discussion

This study aimed to adapt an existing questionnaire and assess the validity and reliability of a self-administered questionnaire. The questionnaire was designed to explore the attitudes, behaviours, and learning resources of rugby coaches and players regarding the ruck event. This questionnaire holds the potential to provide valuable insights into the ruck event, offering a unique perspective that can inform coach development, coaching practices, and player development. The second phase of the study involved a validity assessment through an expert panel, revealing encouraging outcomes regarding the questionnaire. The expert panel reported that the questionnaire was exceptionally clear and devoid of any ambiguities in the questions. This is an important observation as it reflects the careful design of the questionnaire, ensuring it is easily comprehensible and efficiently administered. Furthermore, the mean time required to complete the questionnaire, approximately 22 minutes, indicates the practicality of the questionnaire, as it does not burden the participants. These findings set a solid foundation for the questionnaire’s reliability and practicality.

The ICC Agreement scores for both players and coaches revealed substantial agreement, with players achieving an ICC Agreement score of 0.71 and coaches achieving an even higher score of 0.88. These findings indicate a strong agreement on how participants from both groups responded to the questionnaire questions, an essential aspect of questionnaire reliability. The associated 95% CIs provide a degree of certainty about the likely range for the true ICC values, further underscoring the instrument’s reliability. Additionally, the ICC Consistency scores for both players and coaches, with values of 0.69 for players and 0.87 for coaches, reflect high response consistency and reliability. The narrow CIs surrounding these scores signify that the questionnaire consistently produced reliable responses that were not influenced by random variation. These ICC Agreement and ICC Consistency scores indicate the questionnaire’s stability, reinforcing its suitability for continued research use. Both players and coaches achieved high Kendall’s W scores (0.85 and 0.93, respectively), indicating strong agreement in their rankings. The associated F-statistics and low p-values confirmed the statistical significance of these agreements, implying that the agreement in rankings was not due to random chance. This highlights the robustness of the questionnaire in consistently producing reliable results that genuinely reflect agreement among participants.

It is important to note that the development and validation of this questionnaire followed rigorous methodologies, including expert panel assessments for content validity and test-retest reliability using ICC and Kappa coefficients. The adapted questionnaire, categorised into ‘Training’, ‘Match’, and ‘Learning Resources’, investigates different aspects of the ruck. Training questions assess the importance of rucking, explore technique acquisition, and gather insights on training frequency and intensity. They also evaluate the significance of proper ruck technique, team training frequency, and coaching reflections from the previous season. Match questions focus on factors influencing ruck entry, changes in importance under varying match conditions, and key factors affecting ruck performance and injury prevention. In Learning Resources, the questionnaire gauges time invested in learning techniques and the impact of resources such as playing experience, coaching, workshops, and media. This comprehensive approach aims to unveil valuable insights into coaches’ and players’ experiences, preferences, and perspectives about the ruck. Table 4. presents implementation steps and proposed interventions indicating that using a questionnaire like this can serve as a valuable tool for gathering nuanced feedback, fostering communication, and tailoring injury prevention strategies to the specific needs and perceptions of coaches and players.

### Limitations and future studies

The study’s strength lies in its novelty, being the first to explore the attitudes, behaviours, and learning resources of rugby coaches and players towards the ruck event. However, the potential influence of initial ratings on the test-retest reliability limits the study, as responses on the second occasion may be impacted by the first measurement, potentially undermining the assumption of independence. Another limitation of this study is the potential for sample bias, as including coaches and players from outside of South Africa might introduce certain biases. Nevertheless, this decision was necessary to avoid restricting the sample size in the context of a broader study encompassing coaches and players at different levels within South Africa. Additionally, the recruitment of participants through a social media drive could have contributed to bias in the sample. Despite these limitations, the study makes an important contribution to rugby research and has the potential to inform coach development, coaching practices, and player development. Future studies can explore the implementation of the questionnaire across different countries, from grassroots to professional levels. Another potential area of investigation should examine how this questionnaire compares to existing tools in terms of efficiency, comprehensiveness, and user-friendliness. This questionnaire will enable researchers to understand the attitudes, behaviours, and learning resources within various rugby organisations and setups, encompassing varied budgets. Additionally, it will shed light on how players and coaches perceive the importance of safety during rucking events in rugby.

## Conclusion

In conclusion, this research article highlights the clarity, reliability, and consistency of the questionnaire within a target population of players and coaches. The combined evidence from the ICC Agreement and Consistency scores, alongside Kendall’s W scores and associated statistics, collectively confirm the validity and reliability of the questionnaire as an invaluable research tool. These findings instil confidence in the data collected using this questionnaire, making it a dependable resource for drawing meaningful conclusions and informed decisions in future research in rugby coaching and player development.

## Figures and Tables

**Table 1:** Likert scale responses for validation criteria by the expert panel (n=12)

Validation criteria	Likert scale responses			

	7	8	9	10

Content	1(8)		10(83)	1(8)
Construct		1(8)	9(75)	2(17)
Clarity		1(8)	10(83)	1(8)
Relevance			10(83)	2(17)

Data are presented as *frequency (%)*. The Likert scale responses by the expert panel members ranged between 7 and 10 for all the criteria with a higher number indicating increased relevance.

**Table 2:** Intraclass correlation coefficient (ICC) scores for agreement and consistency among players (n=16) and coaches (n=15)

Stakeholder	ICC Agreement	ICC Agreement (95% CI)	ICC Consistency	ICC Consistency (95% CI)

Players	0.71	0.69–0.73	0.71	0.69–0.73
Coaches	0.88	0.87–0.89	0.87	0.88–0.89

CI, Confidence Interval

**Table 3:** The Kendal W scores for the agreement among players (n=16) and coaches (n=15)

Stakeholder	Kendal W	F-statistic	p-value

Players	0.85	5.66	<0.01
Coaches	0.88	0.87–0.89	<0.01

**Table 4:** Potential implementation steps and proposed interventions following questionnaire research findings

Implementation steps	Interventions
Identifying risk factors	Analyse questionnaire data to identify specific aspects of training and match play perceived as potential risk factors by coaches and players. Collaborate with sports scientists to validate and prioritise these identified risk factors based on existing literature and injury data. Develop targeted interventions to mitigate the identified risks, such as modifications to training drills, equipment enhancements, or rule adjustments. Communicate risk factors and proposed interventions to coaches and players through workshops and training sessions.
Communication and feedback	Analyse feedback related to coaching methods and player experiences, identifying areas for improvement or adjustments. Facilitate structured feedback sessions between coaches and players to discuss findings, fostering open communication and collaborative problem-solving. Implement changes to coaching methods based on feedback, emphasising two-way communication and ongoing dialogue to enhance the coach-player relationship.
Customised training programs	Evaluate perceptions of learning resources to understand player needs and preferences. Collaborate with coaches to tailor training programs based on the questionnaire findings, addressing specific preferences and ensuring adequacy of resources. Integrate player input into the design of training sessions, focusing on areas identified in the questionnaire to minimise the risk of injuries.
Player well-being	Assess player perceptions of their coaches and training environment, identifying elements contributing to wellbeing. Implement strategies to enhance the positive aspects of the training environment, emphasising mental and physical resilience. Provide resources and support for stress management and mental well-being, contributing to an overall positive and supportive atmosphere.
Educational opportunities	Examine questionnaire insights to identify areas where additional education or resources are needed. Develop targeted educational initiatives, including workshops, seminars, or informational materials, to address knowledge gaps and enhance injury prevention awareness. Disseminate educational materials regularly and incorporate educational sessions into the training schedule to ensure ongoing player awareness.

## References

[1] Till K, Hendricks S, Scantlebury S (2023). A global perspective on collision and non-collision match characteristics in male rugby union: Comparisons by age and playing standard. Eur J Sport Sci.

[2] Den Hollander S, Lambert M, Jones B (2019). Tackle and ruck technique proficiency within academy and senior club rugby union. J Sports Sci.

[3] Eime R, Owen N, Finch C (2004). Protective eyewear promotion: Applying principles of behaviour change in the design of a squash injury prevention programme. Sports Med.

[4] Paul L, Davidow D, Stodter A (2023). More than rugby: A scoping review of coaches in rugby. Int J Sports Sci Coach.

[5] Gianotti S, Quarrie K, Hume P (2009). Evaluation of RugbySmart: A rugby union community injury prevention programme. J Sci Med Sport.

[6] Hendricks S, Sarembock M (2013). Attitudes and behaviours of top-level junior rugby union coaches towards the coaching of proper contact technique in the tackle – A pilot study. Afr J Sports Med.

[7] Gilbert W, Lichtenwaldt T, Gilbert J (2009). Developmental profiles of successful high school coaches. Int J Sports Sci Coach.

[8] Hendricks S, Hollander S, Tam N (2015). The relationships between rugby players’ tackles attitudes and behaviour and their match tackle attitudes and behaviour. BMJ Open Sport Exerc Med.

[9] Mountjoy M, Costa A, Budgett R (2018). Health promotion through sport: international sport federations’ priorities, actions and opportunities. Br J Sports Med.

[10] Hendricks S, Jordaan E, Lambert M (2012). Attitude and behaviour of junior rugby union players towards tackling during training and match play. Saf Sci.

[11] Cushion C, Ford PR, Williams AM (2012). Coach behaviours and practice structures in youth soccer: implications for talent development. J Sports Sci.

[12] Orth D, Davids K, Seifert L, Araújo D (2017). Effect of a field-based player-coach training program on the skill performance of youth soccer players. Int J Sports Sci Coach.

[13] Williams AM, Ford PR (2009). Expertise and expert performance in sport. Int Rev Sport Exerc Psychol.

[14] Farrow D, Abernethy B (2003). Do expertise and the degree of perception-action coupling affect natural anticipatory performance?. Perception.

[15] Tsang S, Royse CF, Terkawi AS (2017). Guidelines for developing, translating and validating a questionnaire in perioperative and pain medicine. Saudi J Anaesth.

[16] Haukas A, Storto A, Tiurikova I (2021). Developing and validating a questionnaire on young learners’ multilingualism and multilingual identity. Lang Learn J.

[17] Simoes L, Teixeira-Salmela LF, Magalhaesm L (2018). Analysis of test-retest reliability, construct validity, and internal consistency of the Brazilian version of the pelvic girdle questionnaire. J Manipulative Physiol Ther.

[18] Taber KS (2018). The use of Cronbach’s Alpha when developing and reporting research instruments in science education. Res Sci Educ.

[19] Artino AR, La Rochelle JS, Dezee KJ (2014). Developing questionnaires for educational research: AMEE guide no 87. Med Teach.

[20] Azraii AB, Ramli AS, Ismail Z (2021). Validity and reliability of an adapted questionnaire measuring knowledge, awareness and practice regarding familial hypercholesterolaemia among primary care physicians in Malaysia. BMC Cardiovasc Disord.

[21] Vale L, Fernandes T (2018). Social media and sports: Driving fan engagement with football clubs on Facebook. J Strateg.

[22] El Hajjar ST (2018). Statistical analysis: Internal-consistency, reliability, and construct validity. Int J Quan Qual Res.

[23] Kottner J, Audige L, Brorson S (2011). Guidelines for reporting reliability and agreement studies (GRRAS) were proposed. Int J Nurs Stud.

[24] Shrout PE, Fleiss JL (1979). Intraclass correlations: Uses in assessing rater reliability. Psychol Bull.

[25] Viera AJ, Garret JM (2005). Understanding interobserver agreement: The kappa statistic. Fam Med.

